# Systematic review of near-infrared spectroscopy determined cerebral oxygenation during non-cardiac surgery

**DOI:** 10.3389/fphys.2014.00093

**Published:** 2014-03-17

**Authors:** Henning B. Nielsen

**Affiliations:** Department of Anesthesia, Rigshospitalet, University of CopenhagenCopenhagen, Denmark

**Keywords:** tissue oxygenation, brain, muscle, cerebral cortex, intraoperative monitoring

## Abstract

Near-infrared spectroscopy (NIRS) is used to monitor regional cerebral oxygenation (rScO_2_) during cardiac surgery but is less established during non-cardiac surgery. This systematic review aimed (i) to determine the non-cardiac surgical procedures that provoke a reduction in rScO_2_ and (ii) to evaluate whether an intraoperative reduction in rScO_2_ influences postoperative outcome. The PubMed and Embase database were searched from inception until April 30, 2013 and inclusion criteria were intraoperative NIRS determined rScO_2_ in adult patients undergoing non-cardiac surgery. The type of surgery and number of patients included were recorded. There was included 113 articles and evidence suggests that rScO_2_ is reduced during thoracic surgery involving single lung ventilation, major abdominal surgery, hip surgery, and laparoscopic surgery with the patient placed in anti-Tredelenburg's position. Shoulder arthroscopy in the beach chair and carotid endarterectomy with clamped internal carotid artery (ICA) also cause pronounced cerebral desaturation. A >20% reduction in rScO_2_ coincides with indices of regional and global cerebral ischemia during carotid endarterectomy. Following thoracic surgery, major orthopedic, and abdominal surgery the occurrence of postoperative cognitive dysfunction (POCD) might be related to intraoperative cerebral desaturation. In conclusion, certain non-cardiac surgical procedures is associated with an increased risk for the occurrence of rScO_2_. Evidence for an association between cerebral desaturation and postoperative outcome parameters other than cognitive dysfunction needs to be established.

With the introduction of near infrared spectroscopy (NIRS) for intraoperative evaluation of regional cerebral oxygenation (rScO_2_), focus on maintaining cerebral blood flow (CBF) has lead to intervention algorithms to support cardiac stroke volume and central venous oxygen saturation in addition to mean arterial pressure (MAP), arterial hemoglobin O_2_ saturation, and arterial carbon dioxide pressure (Bundgaard-Nielsen et al., 2007a). Several commercial NIRS-devices provide for a cerebral oximetry evaluation of rScO_2_ reflecting changes in CBF (Madsen and Secher, 1999). During cardiac surgery NIRS is used for anesthetic management of the circulation (Murkin and Arango, 2009) while, as indicated by the number of review papers there is no standard recommendation for the use of NIRS in non-cardiac surgical procedures other than in carotid endarterectomy (CEA; ref. Pennekamp et al., 2009, 2011). In non-cardiac surgery hypotension and in turn a decrease in rScO_2_ may arise when the blood loss challenges the central blood volume or when it is compromised during head-up tilt (Madsen et al., 1995) as used for both abdominal and orthopedic surgery. Thus rScO_2_ may decrease when pressure is reduced below the lower limit of cerebral autoregulation as during cardiac surgery requiring cardiopulmonary by-pass (Ono et al., 2013). Maintained regional tissue blood flow is, however, important for limiting postoperative complications such as acute kidney failure (Chenitz and Lane-Fall, 2012), wound infection (Sørensen, 2012), and cognitive dysfunction (Murkin et al., 2007; Slater et al., 2009) both in cardiac and non-cardiac surgery.

A systematic review was undertaken (i) to determine the non-cardiac surgical procedures that provoke a reduction in rScO_2_ and (ii) to evaluate whether an intraoperative reduction in rScO_2_ influences postoperative outcome such as cognitive dysfunction. Publications included for the review are presented in a table with inclusion of the surgical speciality, the number of patients included in each article, the NIRS device used, and whether cerebral oxygenation was changed intraoperatively.

## Methods

Relevant publications were found by searching the PubMed and Embase database from inception through April 30, 2013. The search strategy combined the following MeSH (medical subject headings) terms and keywords: (NIRS or NIS or near infrared spectroscopy or oximetry), (oximetry or saturation or oxygenation or desaturation or oxygen), (brain or cerebral or muscle), and (surgery or surgical or perioperative).

**Figure 1:**
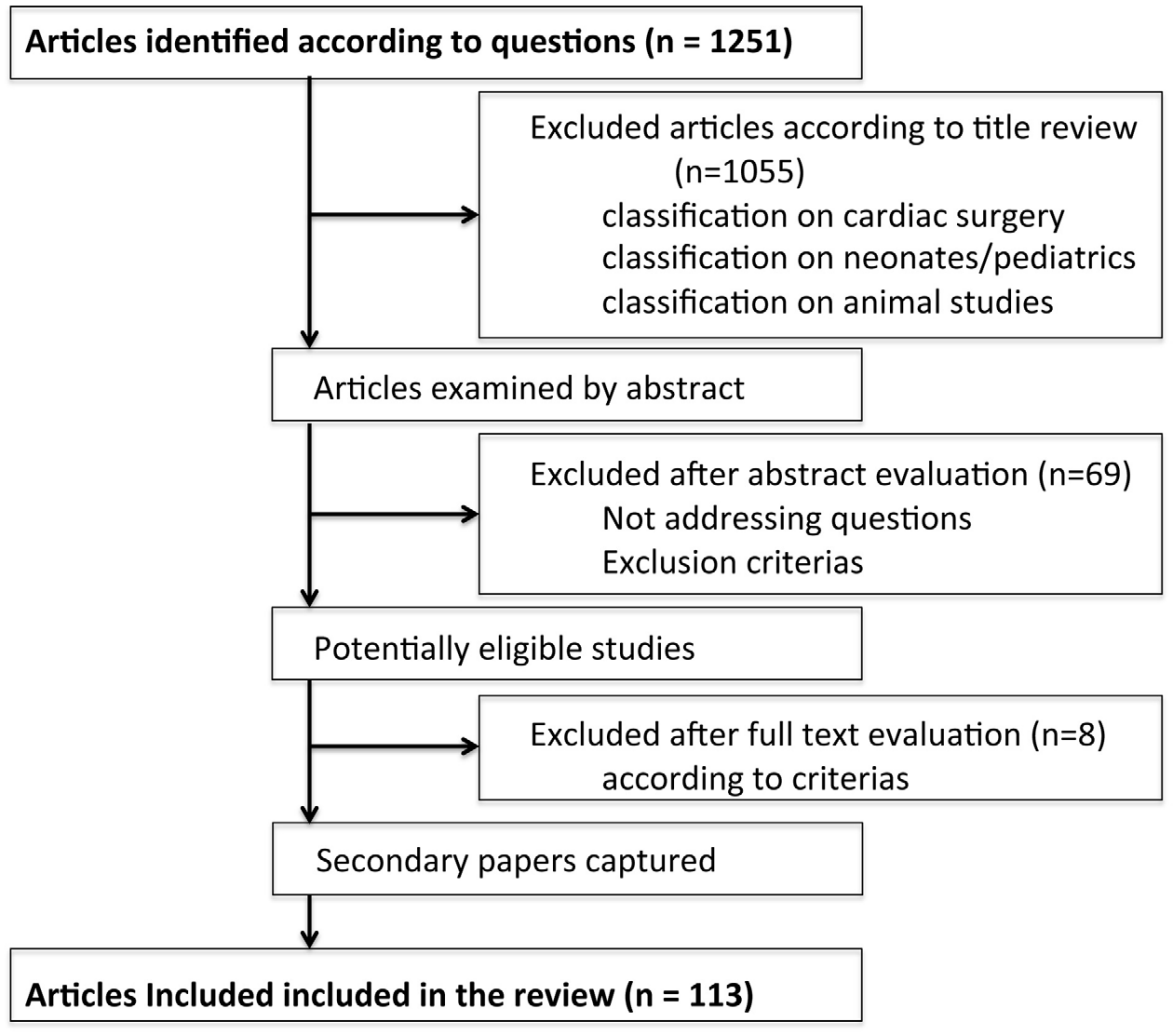
**Flow chart for study selection**.

Publications were included in the review if they addressed monitoring of tissue oxygenation by NIRS for intraoperative monitoring during non-cardiac and non-head-trauma surgery in adult patients (Figure 1). Each title and/or abstract identified was screened for eligibility. Publications were excluded if they did not include original data (e.g., review, commentary), or if they were not published as a full-length article in a peer-reviewed journal. Non-English articles were also excluded and articles evaluating non-brain tissue only were excluded as well. If articles included animals, pediatric patients or cardiac surgical patients they did not fulfill inclusion criteria and they were therefore not considered eligible for inclusion in the study. Articles reporting changes in rScO_2_ before or after surgery were also excluded. Data regarding the number of patients, type of surgery, and type of NIRS for determination of cerebral oxygenation were noted. The articles were grouped according to the predominant surgical procedure.

## Results

Figure 1 is a summary of the search with the initial strategy resulting in 1251 citations. According to title review, 1055 papers did not met the inclusion criteria: 321 papers were on cardiacthoracic and/or pediatric/fetal issues, 54 articles addressed studies in animals, and 99 papers were reviews and/or comments predominantly addressing cardiac surgical patients, 67 articles included head-trauma or neurological patients, 149 articles were in non-English language and 145 papers did not address intraoperative issues. In total 196 articles were included for abstract review. Additional 69 abstracts were excluded for not meeting the main inclusion criteria of this review. After full review additional papers were excluded. NIRS results from 113 papers are presented (Table 1).

**Table 1:** **Studies included in the systematic review grouped in accordance to surgical procedures**.

**Paper**	**Patients**	**Apparatus**	**Intended tissue**	**Change in oxygenation**
**NEUROSURGERY AND SPINE SURGERY**				
Asgari et al., 2003	*N* = 20	Multiscan OS 30	Cortical surface	∧
	Cerebral arteriovenous malformations			
Calderon-Arnulphi et al., 2007	*N* = 25	Oxiplex	Brain	∨
	Neurovascular procedures			
Fuchs et al., 2000	*N* = 74	INVOS 4100	Frontal lobe	∨
	Lumbar discectomy			
	Healthy volunteers			
	CEA			
Lovell et al., 2000	*N* = 20	NIRO 500	Frontal lobe	∨
	Micro discectomy			
	Healthy volunteers			
Paisansathan et al., 2007	*N* = 13	Oxiplex	Frontal lobe	∧
	Spinal or peripheral nerve surgery			
**MAXILLO-FACIAL-EYE SURGERY**				
Choi et al., 2008	*N* = 60	INVOS 5100	Frontal lobe	∨
	Orthognathic surgery			
Fodale	*N* = 66	INVOS 5100B	Frontal lobe	∨
	Ophthalmic procedures			
**BREAST SURGERY**				
Nissen et al., 2009a	*N* = 71	INVOS	Frontal lobe^*^	∧
	mastectomy, thyroidectomy or parathyroidectomy		Skeletal muscle	
Nissen et al., 2010	*N* = 78	INVOS	Frontal lobe^*^	∨
	Mastectomy, thyroidectomy or parathyroidectomy			
**THORACIC SURGERY**				
Tobias et al., 2008	*N* = 40	INVOS 3100A	Frontal lobe	∨
	Open thoracotomy and thorascopy			
Hemmerling et al., 2008	*N* = 20	FORE-SIGHT	Frontal lobe	∨
	Open thoracotomy			
Kazan et al., 2009	*N* = 50	FORE-SIGHT	Frontal lobe	∨
	Thoracic surgery			
Tang et al., 2012	*N* = 76	FORE-SIGHT	Frontal lobe	∨
	Thoracic surgery			
**ORTHOPEDIC SURGERY**				
Dippmann et al., 2010	*N* = 2	INVOS 5100	Frontal lobe	∨
	Arthroscopic shoulder surgery			
Fischer et al., 2009	*N* = 1	FORESIGHT	Frontal lobe	∨
	Arthroscopic shoulder surgery			
Jeong et al., 2012	*N* = 56	INVOS 5100B	Frontal lobe	∨
	Arthroscopic shoulder surgery			
Han et al., 2006	*N* = 56	INVOS 4100	Frontal lobe	∨
	Major orthopedic surgery			
Lee et al., 2011	*N* = 28	INVOS 5100	Frontal lobe	∨
	Arthroscopic shoulder surgery			
Lin et al., 2013	*N* = 46	INVOS 5100B	Frontal lobe	∨
	Total hip arthroplasty			
Ko et al., 2012	*N* = 50	INVOS 5100	Frontal lobe	∨
	Arthroscopic shoulder surgery			
Moerman et al., 2012	*N* = 20	INVOS 5100	Frontal lobe	∨
	Arthroscopic shoulder surgery			
Murphy et al., 2010	*N* = 124	FORE-SIGHT	Frontal lobe	∨
	Arthroscopic shoulder surgery in beach chair and LDP			
Papadopoulos et al., 2012	*N* = 69	INVOS 5100C	Frontal lobe	∨
	Hip fracture repair			
Salazar et al., 2013a	*N* = 51	INVOS 5100	Frontal lobe	∨
	Arthroscopic shoulder surgery			
Salazar et al., 2013b	*N* = 50	INVOS 5100	Frontal lobe	∨
	Arthroscopic shoulder surgery			
Song et al., 2012	*N* = 28	INVOS 5100	Frontal lobe	–
	Total knee replacement			
Tange et al., 2010	*N* = 30	NIRO-200	Frontal lobe	–
	Arthroscopic shoulder surgery			
Tzimas et al., 2010	*N* = 1	INVOS 5100	Frontal lobe	∧
	Hip fracture repair			
Yadeau et al., 2011	*N* = 99	INVOS 5100C	Frontal lobe	∨
	Arthroscopic shoulder surgery			
Yoshitani et al., 2005	*N* = 42	INVOS 4100	Frontal lobe	∨
	Total hip arthroplasty			
**UROLOGY**				
Bundgaard-Nielsen et al., 2007b	*N* = 12	INVOS	Frontal lobe	–
	Open prostatectomy		Biceps muscle	
Burkhart et al., 2011	*N* = 104	NIRO-200	Frontal lobe	∨
	Non-epidural major surgery			
Kalmar et al., 2012	*N* = 31	FORE-SIGHT	Frontal lobe	–
	Robot prostatectomy			
Meng et al., 2012	*N* = 29	Oxiplex	Frontal lobe	∨
	Predominant patients for robot prostatectomy			
Meng et al., 2011	*N* = 14	Oxiplex	Frontal lobe	∨
	Predominant patients for robot prostatectomy			
Park et al., 2009	*N* = 32	INVOS 5100	Frontal lobe	–
	Robot prostatectomy			
**GYNECOLOGY**				
Berlac and Rasmussen, 2005	*N* = 38	INVOS 3100	Frontal lobe	∨
	Caesarean section			
Fassoulaki et al., 2006	*N* = 44	INVOS 3100	Frontal lobe	∨
	Hysterectomy			
Kondo et al., 2013	*N* = 42	NIRO pulse	Brain	∨
	Caesarean section			
Lee et al., 2006	*N* = 24	INVOS 4100	Frontal lobe	∨
	Laparoscopic gynecology			
Morimoto et al., 2000	*N* = 45	NIRO-500	Frontal lobe	∧
	Gynecologic surgery			
**GASTRO-INTESTINAL SURGERY**				
Casati et al., 2005	*N* = 122	INVOS 4100	Frontal lobe	∨
	Major abdominal surgery			
Casati et al., 2007	*N* = 60	INVOS 4100	Frontal lobe	∨
	Major abdominal surgery			
Gipson et al., 2006	*N* = 70	INVOS 3100A	Frontal lobe	∨
	Laparoscopic herniorrhaphy, cholecystectomy, gastric bypass			
Green, 2007	*N* = 46	INVOS	Frontal lobe	∨
	Major abdominal surgery: whipple, hepatectomy, prostatectomy, cystectomy, aortic aneurysm repair			
Harrison, 2001	*N* = 13	INVOS 3100	Frontal lobe	∨
	Surgery for gastrointestinal or gynecological malignancy			
Kitajima et al., 1998	*N* = 12	NIRO-500	Brain	∨
	Laparoscopic cholecystectomy			
Kurukahvecioglu et al., 2008	*N* = 60	INVOS 5100	Frontal lobe	∨
	Laparoscopic cholecystectomy			
Madsen et al., 2000	*N* = 48	INVOS 3100	Frontal lobe	∨
	Liver transplantation			
Madsen and Secher, 2000	*N* = 1	INVOS 3100	Frontal lobe	∨
	Liver transplantation			
Morimoto et al., 2009	*N* = 20	INVOS 3100	Frontal lobe	∨
	Laparotomy or laparoscopic surgery			
Nissen et al., 2009b	*N* = 33	INVOS	Frontal lobe	∨
	Liver transplantation			
Plachky et al., 2004	*N* = 16	INVOS 3100A	Frontal lobe	∨
	Liver transplantation			
Zheng et al., 2012	*N* = 9	INVOS	Frontal lobe	∨
	Liver transplantation	(Somanetics)		
**VASCULAR SURGERY**				
Liu et al., 1999^††^	*N* = 12	INVOS-3100	Frontal lobe	∨
	AAA patients			
Kuroda et al., 1996a	*N* = 5	OM-100	Frontal lobe	∨
	Balloon occlusion test of ICA	(Shimadzu Co.)		
Torella et al., 2002^**^	*N* = 30	INVOS-4100	Frontal lobe	∨
	Aortic surgery		Calf muscle	
Torella et al., 2003^***^	*N* = 29	INVOS-4100	Frontal lobe	∧
	Aortic surgery (*n* = 21)		Calf muscle	
Torella and McCollum, 2004^****^	Spinal surgery (*n* = 8)	INVOS-4100	Frontal lobe	
			Calf muscle	
**CAROTID SURGERY**				
Ali et al., 2011	*N* = 10	INVOS	Frontal lobe	∨
	Aortic surgery			
Beese et al., 1998	*N* = 49	INVOS-3100	Frontal lobe	∨
	CEA, LA			
Carlin et al., 1998	*N* = 137	INVOS-3100	Frontal lobe	∨
	CEA, GA			
Cho et al., 1998	*N* = 16	INVOS-3100A	Frontal lobe	∨
	CEA, LA	NIRO500 (*n* = 20)		
Cuadra et al., 2003	*N* = 29	INVOS-4100	Frontal lobe	∨
	CEA, GA			
Duncan et al., 1995	*N* = 40	–	Frontal lobe	∨
	CEA, GA			
Duffy et al., 1997	*N* = 22	INVOS-3100	Frontal lobe	∨
Espenell et al., 2010	*N* = 72	FORE-SIGHT	Frontal lobe	∨
	CEA, GA			
Fassiadis et al., 2006	*N* = 35	INVOS	Frontal lobe	∨
	CEA, GA			
Fearn et al., 2000	*N* = 40	INVOS-3100A	Frontal lobe	∨
	CEA, LA			
Friedell et al., 2008	*N* = 100	INVOS	Frontal lobe	∨
	CEA			
Giustiniano et al., 2010	*N* = 323	INVOS-5100B	Frontal lobe	∨
	CEA, GA			
Grubhofer et al., 1997	*N* = 104	INVOS-3100A	Frontal lobe	∨
	CEA, GA			
Grubhofer et al., 2000	*N* = 12	INVOS-3100	Frontal lobe	∨
	CEA, GA			
Ishigaki et al., 2008	*N* = 59	TOS96	Frontal lobe	∨
	CEA, GA			
Kacprzak et al., 2012	*N* = 41	Selfconstruct	Frontal lobe	∨
	CEA, GA			
Kawada et al., 2002	*N* = 16	TOS	Frontal lobe	∨
	CEA			
Kobayashi et al., 2009	*N* = 3	TOS96	Frontal lobe	∨
	Extracranial ICA			
	Aneurysm			
Komoribayashi et al., 2006	*N* = 171	TOS96	Frontal lobe	∨
	CEA, GA			
Kragsterman et al., 2004	*N* = 89	INVOS4100	Frontal lobe	∨
	CEA, GA			
Kuroda et al., 1996b	*N* = 62	OM100/110	Frontal lobe	∨
	CEA, GA			
Laffey et al., 2000	*N* = 22	INVOS3100	Frontal lobe	∨
	CEA, GA			
Lee et al., 2008	*N* = 1	INVOS4100	Frontal lobe	∨
	CEA, GA			
de Letter et al., 1998	*N* = 37	–	Frontal lobe	∨
	CEA, GA			
McCleary et al., 1996	*N* = 102	Critikon	Frontal lobe	∨
	CEA, GA			
Manwaring et al., 2010	*N* = 65	INVOS	Frontal lobe	∨
	CEA, LA/GA			
Mason et al., 1994	*N* = 104	NIRO500	Frontal lobe	∨
	CEA, GA			
Mead et al., 1996	*N* = 11	INVOS	Frontal lobe	∨
	CEA, GA			
Matsumoto et al., 2009	*N* = 16	INVOS5100	Frontal lobe	∨
	CEA			
Mille et al., 2004	*N* = 64	INVOS 3100/4100	Frontal lobe	∨
	CAS, LA			
Moritz et al., 2007	*N* = 594	INVOS3100	Frontal lobe	∨
	CEA, GA			
Moritz et al., 2010	*N* = 48	INVOS3100	Frontal lobe	∨
	CEA, LA			
Nakamura et al., 2009	*N* = 96	INVOS3110A/OMM2000	Frontal lobe/Global brain	∨
	CEA, LA/GA			
Ogasawara et al., 2003	*N* = 1	TOS96	Frontal lobe	∨
	CEA			
Pedrini et al., 2012	*N* = 50	INVOS4100	Frontal lobe	∨
	CEA, GA			
Pennekamp et al., 2012a	*N* = 473	INVOS	Frontal lobe	∨
	CEA, GA			
Pennekamp et al., 2012b	*N* = 11	INVOS	Frontal lobe	∨
	CEA, GA			
Pugliese et al., 2009	*N* = 151	INVOS	Frontal lobe	∨
	CEA, GA			
Rigamonti et al., 2005	*N* = 40	INVOS4100	Frontal lobe	∨
	CEA, LA			
Ritter et al., 2011	*N* = 50	INVOS4100	Frontal lobe	∨
	CEA, LA			
Samra et al., 1996	*N* = 83	INVOS3100	Frontal lobe	∨
	CEA, LA			
Samra et al., 2000	*N* = 38	INVOS3100	Frontal lobe	∨
	CEA, LA			
Samra et al., 1999	*N* = 99	INVOS3100	Frontal lobe	∨
	CEA, LA			
Sehic and Thomas, 2000	*N* = 34	INVOS3100A	Frontal lobe	∨
	CEA, LA			
Shang et al., 2011	*N* = 1	DCS flow-oximeter	Frontal lobe	∨
	CEA, GA			
Stilo et al., 2012	*N* = 11	INVOS4100	Frontal lobe	∨
	CEA, GA			
Stoneham et al., 2008	*N* = 100	INVOS4100	Frontal lobe	∨
	CEA, LA			
Takeda et al., 2000	*N* = 16	INVOS3100	Frontal lobe	∨
	CEA, LA			
Tambakis et al., 2011	*N* = 24	INVOS4100	Frontal lobe	∨
	CEA			
Uchino et al., 2012	*N* = 56	INVOS5100C	Frontal lobe	∨
	CEA, GA			
Vets et al., 2004	*N* = 20	NIRS	Frontal lobe	∨
	CEA, GA			
Williams et al., 1999	*N* = 14	Critikon2020	Frontal lobe	∨
	CEA			
Yamamoto et al., 2007	*N* = 45	OM-220	Frontal lobe	∨
	CEA, LA			
Zogogiannis et al., 2011	*N* = 43	INVOS4100	Frontal lobe	∨
	CEA, GA			

For changes in oxygenation during vascular surgical procedures see text for specific results. LA, local anesthesia; GA, general anesthesia. The full papers by Williams et al. (1994a,b,c) could not be retrieved. As these papers are among the first to report rScO_2_ in patients undergoing CEA the papers are cited in the text but not in the table.

** Following 30 min acute normovolemic hemodilution decreased tissue oxygenation that reduced the hemoglobin concentration from 14.5 to 10.8 g/dl.

*** Increased tissue oxygenation following blood transfusion.

**** Reduced tissue oxygenation following blood loss equivalent to 650 ml or 16% of the patients' blood volume.

†† Decreased cerebral oxygenation with aortic cross-clamping and following declamping increased oxygenation.

### Neurosurgery and surgery on the spine

During neurovascular procedures (aneurysm clipping, bypass procedures, or balloon occlusion testing), rScO_2_, and the NIRS-determined concentration of oxygenated hemoglobin (HbO_2_) decrease (Calderon-Arnulphi et al., 2007) and rScO_2_ reflects the success of surgical resection of a cerebral arterio-venous malformation (Asgari et al., 2003). While induction of anesthesia does not change brain oxygenation tracheal intubation increases HbO_2_ (Paisansathan et al., 2007). In contrast the head up tilted position provokes a decrease in rScO_2_ (69 vs. 71%) (Fuchs et al., 2000) and also the NIRS-determined total Hb becomes reduced (Lovell et al., 2000).

### Maxillo-facial-eye surgery and breast surgery

Minor reduction in rScO_2_ is observed immediately after peribulbar block for eye surgery (Fodale et al., 2006) and with MAP reduced to 60 mmHg during orthognathic surgery rScO_2_ decreases 5% (Choi et al., 2008). Such changes do not provoke postoperative cognitive dysfunction (POCD) as determined by a decrease in the minimal mental state examination (MMSE) score =2 points from baseline (Choi et al., 2008).

In patients scheduled for mastectomy induction of anesthesia with subsequent hypotension, rScO_2_ increases (from 67 to 72%) to remain stable during surgery (Nissen et al., 2009a, 2010). While ephedrine preserves rScO_2_, phenylephrine is reported to decrease rScO_2_ 14% (Nissen et al., 2010).

### Thoracic surgery

During open thoracotomy or thorascopy, about half of the patients present at least one rScO_2_ value that is lower than 80% of the baseline value (Tobias et al., 2008) and during surgery with single lung ventilation up to 75% of the patients suffer from a more than a 20% decrease in rScO_2_ (Hemmerling et al., 2008; Kazan et al., 2009; Tang et al., 2012). Risk factors for a reduction in rScO_2_ are age, weight, and ASA class III (Tobias et al., 2008) and the minimum rScO_2_ value predicts postoperative complications as evaluated by the Clavien and SOFA scoring systems (Kazan et al., 2009). The exposure time to rScO_2_ values below <65% correlates with occurrence of POCD (Tang et al., 2012). This study used MMSE for evaluation of cognitive function before surgery and several days after surgery. A decrease >2 points from baseline was defined as POCD.

### Shoulder surgery

During arthroscopic shoulder surgery in the lateral decubitus position, rScO_2_ is maintained (Murphy et al., 2010) but when the patient is placed in the beach chair position rScO_2_ may decrease (Fischer et al., 2009; Dippmann et al., 2010; Tange et al., 2010; Lee et al., 2011; Yadeau et al., 2011; Jeong et al., 2012; Ko et al., 2012; Moerman et al., 2012; Salazar et al., 2013a,b) with different incidence of intraoperative cerebral desaturation (0 vs. 27%) (Tange et al., 2010; Jeong et al., 2012). The duration of cerebral desaturation episodes range from 1 min to 1 h or longer (Jeong et al., 2012). In the recent study by Salazar et al. (2013a), it is stated that mean maximal desaturation is 32% with each desaturation event lasting an average of 3 min 3 s. Lowered rScO_2_ coincides with low MAP (<70 mmHg; 30, 33, 36) and raised MAP restores rScO_2_ (Lee et al., 2011). In a case report including one patient it is noted that the α_1_-agonist phenylephrine increases both MAP and rScO_2_ (Fischer et al., 2009). Large body mass index is reported to be associated with a reduction in rScO_2_ (Salazar et al., 2013a).

The influence of intravenous (propofol) anesthesia vs. inhalational (sevoflurane) anesthesia on rScO_2_ has also been evaluated (Jeong et al., 2012). During surgery in the beach chair patients in sevoflurane anesthesia have higher internal jugular venous O_2_ saturation (SjvO_2_) than patients in propofol anesthesia (minimum SjvO_2_ 63 vs. 42%), rScO_2_ is similar in the two groups and rScO_2_ and SjvO_2_ correlate. As MAP also is higher with sevoflurane anesthesia, despite a less frequent use of vasopressors, the authors conclude that sevoflurane anesthesia may be a better choice in patients undergoing surgery in beach chair position (Jeong et al., 2012).

An influence of cerebral desaturation on the occurrence of POCD after shoulder surgery in the beach chair is evaluated by Salazar et al. (2013b). Based on a Repeatable Battery for the Assessment of Neuropsychological Status (RBANS) score the authors conclude that POCD is almost identical in subjects with intraoperative cerebral desaturation compared to those in the cohort who did not (Salazar et al., 2013b). The findings are supported by Moerman et al. (2012) who report that neurological or cognitive dysfunction does not occur after surgery in the beach chair.

### Other types of orthopedic surgery

Major orthopedic surgery (hip surgery) reduces rScO_2_ ≈10% below baseline, with esmolol induced hypotension, rScO_2_ becomes even lower (Han et al., 2006) and also the NIRS-determined deoxygenated hemoglobin (Hb) concentration decreases (Yoshitani et al., 2005). During hip fracture repair rScO_2_ <50 or 75% of baseline occurs in 38% of patients (Papadopoulos et al., 2012) and rScO_2_ decreases independently of the anesthesia used (propofol vs. sevoflurane; ref. Yoshitani et al., 2005). During knee surgery rScO_2_ remains stable (Song et al., 2012).

Before surgery neurocognitive dysfunction is associated to low rScO_2_ (44%) (Tzimas et al., 2010) and in patients with cerebral desaturation during major orthopedic surgery the occurrence of POCD is reported to increase (Papadopoulos et al., 2012; Lin et al., 2013). Following surgery for hip fracture, patients with POCD have lower intraoperative rScO_2_ (55 vs. 65%) compared to non-POCD patients (Papadopoulos et al., 2012). In this study cognitive function was assessed by the MMSE preoperatively and on the 7th postoperative day and compared to baseline, a reduction of MMSE score by >2 points indicated POCD. Lin et al. (2013) used MMSE, digit span test, digit symbol substitution test, trail making test, verbal fluency test, and word recognition tests and it was noted that in patients with POCD the intraoperative rScO_2_ drop (14 vs. 8%) was more marked compared to non-POCD patients (Lin et al., 2013). The authors suggest that an intraoperative decrease in rScO_2_ max >11% is to be considered a warning signal for development of POCD (Lin et al., 2013).

### Urology

In patients undergoing robotic assisted prostactomy in the Trendelenburg position rScO_2_ is reported to increase (Park et al., 2009; Kalmar et al., 2012). However, the elderly patient may demonstrate profound intraoperative desaturation (to 20% or more below baseline) (Burkhart et al., 2011). Also hemodilution may lower rScO_2_ (Bundgaard-Nielsen et al., 2007b) and a reduction in rScO_2_ correlates to development of hypotension (Burkhart et al., 2011). The use of phenylephrine to preserve MAP reduces rScO_2_ and this effect is intensified by hypocapnia and blunted by hypercapnia (Meng et al., 2012). Importantly, rScO_2_ remains unchanged after bolus ephedrine (Meng et al., 2011).

### Gynaecological and obstetric procedures

During gynecological laparoscopic procedures in the Trendelenburg position rScO_2_ decreases from 66 to 57% with MAP at 80 mmHg (Lee et al., 2006). Different gas anesthesia (desflurane vs. sevoflurane) results in similar rScO_2_ values and larger anesthetic depth increases rScO_2_ (66 vs. 72%) (Fassoulaki et al., 2006). Also spinal anesthesia reduces rScO_2_ (>5%) related to development of hypotension (Berlac and Rasmussen, 2005). The use of hyperbaric rather than isobaric bupivacaine for spinal anesthesia decreases HbO_2_ (6 vs. 3 mmol/L) as also hypotension is more severe (Kondo et al., 2013). In contrast, tracheal extubation increases HbO_2_ (Morimoto et al., 2000). The authors also demonstrate that compared with a control nicardipine and diltiazem inhibited an increase in MAP and further enhanced the increase in HbO_2_ (Morimoto et al., 2000).

In a patient with an intraoperative reduction in rScO_2_ to below 50% is reported to be the likely explanation for postoperative headache (Lee et al., 2006).

### Gastro-abdominal surgery

Laparoscopic cholecystectomy in the head-up position is reported to decrease HbO_2_ even when MAP is maintained above 80 mmHg (Kitajima et al., 1998) and up to one-fifth of the patients present at least one rScO_2_ value of less than 80% of baseline (Gipson et al., 2006). Even in the supine position, rScO_2_ tends to be reduced while the head-down position maintains rScO_2_ (Harrison, 2001). A lowered rScO_2_ can be restored by intermittent sequential compression of the lower extremities (Kurukahvecioglu et al., 2008).

A 15% decrease in rScO_2_ correlates with the blood loss (Green, 2007) and in the elderly patient minimum rScO_2_ (49 vs. 55%), mean rScO_2_ (61 vs. 66%) and area under curve rScO_2_ are higher with interventions that improve rScO_2_ (Casati et al., 2005). In liver patients high bilirubin (icterus) interfere with NIRS measurements (Madsen et al., 2000), however, an intraoperative decrease in rScO_2_ by up to 13% correlates to release of neuron-specific enolase (Plachky et al., 2004). NIRS is also used for investigation of cerebral autoregulation during a liver transplantation (Nissen et al., 2009b; Zheng et al., 2012) and rScO_2_ decreases markedly after clamping the caval vein (Plachky et al., 2004).

A possible relationship between intraoperative cerebral desaturation and development of POCD was first described in a case report (Madsen and Secher, 2000). In randomized clinical trial Casati et al. (2005) included a total of 122 patients from 5 participating hospitals randomly allocated to an intervention group (with a NIRS visible and rScO_2_ maintained at =75% of preinduction values) or a control group. No differences in MMSE score were observed. However, at the seventh postoperative day those patients of the control group who had intraoperative desaturation showed lower value of MMSE (26 vs. 28) as compared with patients of the treatment group. Patients of the control group who had intraoperative desaturation also showed a longer hospital stay as compared with patients of the treatment group. These findings were confirmed by another study by Casati et al. (2007) and the authors further report that up to one in every four patients demonstrate cerebral desaturation. Furthermore, in patients with postoperative delirium intraoperative rScO_2_ is lower compared to patients with no delirium (57 vs. 60%; ref. Morimoto et al., 2009).

### Vascular surgery

Open aortic repair of an abdominal aortic aneurysm affects rScO_2_ (Liu et al., 1999) with a reduction in proportion to the blood loss (Torella and McCollum, 2004) and hemodilution (Torella et al., 2002) while blood transfusions increase rScO_2_ (Torella et al., 2003). Several report rScO_2_ during carotid surgery (Williams et al., 1994a,b,c, 1999; Duncan et al., 1995; Kuroda et al., 1996a; Mead et al., 1996; Samra et al., 1996; Duffy et al., 1997; Beese et al., 1998; Carlin et al., 1998; de Letter et al., 1998; Fearn et al., 2000; Takeda et al., 2000; Kawada et al., 2002; Cuadra et al., 2003; Ogasawara et al., 2003; Vets et al., 2004; Komoribayashi et al., 2006; Yamamoto et al., 2007; Ishigaki et al., 2008; Lee et al., 2008; Stoneham et al., 2008; Kobayashi et al., 2009; Giustiniano et al., 2010; Moritz et al., 2010; Ali et al., 2011; Ritter et al., 2011; Pedrini et al., 2012; Uchino et al., 2012).

During CEA clamping the internal carotid artery (ICA) decreases ipsilateral rScO_2_ (Williams et al., 1994a,b,c, 1999; Duncan et al., 1995; Mead et al., 1996; Samra et al., 1996; Duffy et al., 1997; Beese et al., 1998; Carlin et al., 1998; de Letter et al., 1998; Fearn et al., 2000; Takeda et al., 2000; Cuadra et al., 2003; Ogasawara et al., 2003; Vets et al., 2004; Komoribayashi et al., 2006; Yamamoto et al., 2007; Ishigaki et al., 2008; Lee et al., 2008; Stoneham et al., 2008; Kobayashi et al., 2009; Giustiniano et al., 2010; Moritz et al., 2010; Ali et al., 2011; Ritter et al., 2011; Pedrini et al., 2012; Uchino et al., 2012) corresponding to a drop in HbO_2_ (Kuroda et al., 1996b; Cho et al., 1998; Shang et al., 2011) and the contralateral rScO_2_ remains largely unchanged (Samra et al., 1999). Clamping the external carotid artery may decrease rScO_2_ 1–3% (Kuroda et al., 1996b; Samra et al., 1999; Fearn et al., 2000) and after ICA clamp a decrease in rScO_2_ often exceeds 20% (Pedrini et al., 2012). An influence of anatomic irregularities in skull shape and cerebral venous drainage needs to be considered. In a case report it is described that inability to obtain a monitorable signal may be attributed to abnormal frontal sinus ipsilateral to the endarterectomy site (Sehic and Thomas, 2000). Another factor of importance is that diabetic patients are more likely to demonstrate a drop in rScO_2_ >20% (Stilo et al., 2012).

With clamped ICA a change in rScO_2_ also reflects a change in the transcranial doppler determined cerebral perfusion (Mason et al., 1994; Fearn et al., 2000; Grubhofer et al., 2000; Vets et al., 2004; Fassiadis et al., 2006; Pugliese et al., 2009; Shang et al., 2011) and also in the reperfusion phase changes in rScO_2_ correlate to measures of CBF (Ogasawara et al., 2003; Matsumoto et al., 2009). Similarly, rScO_2_ correlates to SjvO_2_ (Williams et al., 1994b; Grubhofer et al., 1997; Espenell et al., 2010) and a correlation to stump pressure is also reported (Kragsterman et al., 2004; Yamamoto et al., 2007; Lee et al., 2008; Manwaring et al., 2010; Tambakis et al., 2011) so that a low stump (<40 mmHg) results in a large change in rScO_2_ (Tambakis et al., 2011) but the relationship might be absent in a large series of patients (Pedrini et al., 2012). rScO_2_ and systemic blood pressure correlate, with higher pressures leading to better oxygenation values (Williams et al., 1994c; Ritter et al., 2011). The use of multichannel NIRS with 8 lightsource fibers and 8 detectors providing 24 source-detector pairs supports that following application of ICA cross clamp, HbO_2_, and Hb change in the border region between the right middle and posterior cerebral supply areas (Nakamura et al., 2009) with distinct changes in Hb and HbO_2_ of the ipsilateral brain cortex (Kacprzak et al., 2012).

Oxygen breathing (Stoneham et al., 2008) and the use of ephedrine (Pennekamp et al., 2012a) increase rScO_2_ while it declines following administration of phenylephrine (Pennekamp et al., 2012a). The most effective approach to increase rScO_2_ during CEA, however, is to use a shunt (Cuadra et al., 2003; Ali et al., 2011; Ritter et al., 2011; Pedrini et al., 2012). Especially patients with rScO_2_ drop >20% require shunting (Ritter et al., 2011; Stilo et al., 2012) and NIRS has a sensitivity of ≈75% and specificity ≈98% of the need for shunting (Ali et al., 2011; Ritter et al., 2011). The criterion for establishing a shunt is (i) a 20% drop in ipsilateral rScO_2_ from baseline (Zogogiannis et al., 2011) or (ii) a change in rScO_2_ greater than 25% or a delta rScO_2_ greater than 20% that is not improved within 3 min by increasing blood pressure (Pedrini et al., 2012), or (iii) a cut-off of 21 or 10% reduction from the baseline (Tambakis et al., 2011). In patients operated under cover of local anesthesia (LA), it is the awake testing procedure that determines when a shunt is needed (Stilo et al., 2012).

Neurological deterioration relates to a decrease in rScO_2_ (Williams et al., 1999; Samra et al., 2000; Moritz et al., 2007) and the anesthetic approach might be important (McCleary et al., 1996; Moritz et al., 2010). In symptomatic patients rScO_2_ decreases from 63 to 51% compared to a rScO_2_ drop from 66 to 61% in non-symptomatic patients (Williams et al., 1999; Samra et al., 2000). About 10% of patients have neurologic changes after carotid clamping (Moritz et al., 2007). Indices of cerebral ischemia (amplitude transcranial motor evoked potentials, electroencephalographic evaluation, cortical somatosensory evoked potentials) correlate to rScO_2_ (Beese et al., 1998; Rigamonti et al., 2005; Uchino et al., 2012) and rScO_2_ needs to decrease >10% for cerebral ischemia to be detected by somatosensory evoked potentials (Duffy et al., 1997) or electroencephalography (Friedell et al., 2008).

Importantly, in patients with focal cerebral ischemia with an embolic event in the territory of the middle cerebral artery ipsilateral frontal lobe rScO_2_ is unchanged (Laffey et al., 2000). However, a reduction in an ischemic ratio (the lowest rScO_2_ value during clamping of the ICA divided by the mean rScO_2_ value in the last 2 min before ICA clamping) predicts new neurological deficit following CEA (Kobayashi et al., 2009) and a large decrease in intraoperative rScO_2_ reflects a change in cerebral metabolism (Espenell et al., 2010). The cerebral release of matrix metalloproteinase correlates to development of cerebral ischemia as determined by NIRS (Ishigaki et al., 2008). rScO_2_ criteria for cerebral ischemia is (i) a rScO_2_ drop of 10 index points from a stable baseline (ii) a rScO_2_ decrease below an absolute value of 50%, (iii) a relative rScO_2_ decrease by 20–25%, and (iv) an interhemispheric rScO_2_ difference of >25% (Friedell et al., 2008). Using NIRS during CEA neurologica deficit is predicted 5–10 s before the clinical observation of neurological complications (Pugliese et al., 2009).

Postoperative neurological complications may rise following an early drop in rScO_2_ by more than 20% (Mille et al., 2004) and rScO_2_ reduction of at least 15% relates to neurologic, cardiac or renal postoperative complications (Rigamonti et al., 2005; Giustiniano et al., 2010). Thus a fall of larger than 10% from baseline rScO_2_ is dangerous but less than 5% is safe (Takeda et al., 2000). The postoperative cerebral hyperperfusion syndrome (CHS) can also be predicted by the intraoperative change in rScO_2_ during clamping and unclamping ICA (Cho et al., 1998; Komoribayashi et al., 2006). After declamping a change in rScO_2_ >20% predicts CHS (Pennekamp et al., 2012b) and patients with CHS exhibit a larger increase in rScO_2_ (Matsumoto et al., 2009).

## Discussion

The present study aimed (i) to determine the non-cardiac surgical procedures that provoke a reduction in rScO_2_ and (ii) to evaluate whether an intraoperative reduction in rScO_2_ influences postoperative outcome. A literature search was conducted and several articles were reviewed. The Results section provides an overview of different non-cardiac surgical procedures affecting rScO_2_ and the included articles representing case reports, observational studies, interventional studies, and randomized clinical trials with inclusion of single patients up to a population of 594 patients. The studies also differ in terms of patient categories, interventions applied and the NIRS device used for the evaluation of rScO_2_. Taken the heterogeneous material into consideration the included articles provide answer to the primary aim of the present study. Based on the Results section it is concluded that some but not all non-cardiac surgical procedures may decrease rScO_2_. While rScO_2_ appears to be maintained in patients undergoing minor non-cardiac surgery such as mastectomy, rScO_2_ is reported to decrease during surgery involving procedures such as the anti-Trendelenburg body position often used for shoulder surgery and laparoscopic surgery. Hip surgery, single lung ventilation in thoracic surgery, and clamped ICA also appear to be associated with a reduction of rScO_2_.

Concerning the second aim of the present review, only a limited number of studies report that the occurrence of cerebral desaturation is linked to bad postoperative outcome: (i) a randomized clinical trial including elderly patients for major abdominal surgery suggests that in patients with intraoperative optimization of rScO_2_ the occurrence of POCD and length of stay in hospital become reduced, (ii) a study on patients undergoing thoracic surgery reports an association between low rScO_2_ and scores of postoperative complications, and (iii) low rScO_2_ may predict POCD in patients undergoing thoracic surgery, major orthopedic surgery, and major abdominal surgery. Also in patients undergoing carotid endarterectomy low rScO_2_ coincides with measures of bad outcome: indices of cerebral ischemia during surgery and the occurrence of the CHS after surgery. However, pronounced intraoperative cerebral desaturation does not lead to POCD after shoulder surgery in the beach chair. Furthermore, an association between cerebral desaturation and outcome parameters such as acute kidney failure, postoperative wound infection, myocardial infarction remains to be established. So the overall conclusion is that the available evidence points toward an increase in the occurrence of POCD in patients with severe cerebral desaturation under certain types of non-cardiac surgery but more studies are needed to demonstrate a clear association between low rScO_2_ and bad postoperative outcome.

In the studies supporting a potential association between rScO_2_ and bad postoperative outcome, a 20–25% decline in rScO_2_ appears to predict POCD and in accordance to the reviewed articles the recommendation is that in order to prevent reaching this potentially injurious level, a less extreme threshold of perhaps 10% should be an indicator for therapeutic intervention to raise cerebral O_2_ saturation. Thus, with a NIRS probe attached to the forehead enables the anesthetist to follow changes in regional CBF changes both in local and global cerebral oxygenation can be monitored. The obtained value for tissue oxygenation reflects a balance between O_2_ delivery and extraction measurements. Therefore factors influencing regional blood flow (Madsen and Secher, 1999; Boushel et al., 2001) such as hemoglobin concentration, blood volume, cardiac output, arterial hemoglobin O_2_ saturation, and for the brain arterial carbon dioxide pressure (PaCO_2_) need to be considered when NIRS is incorporated for clinical evaluations. For most of the studies included in the present review it is not obvious how such factors were controlled.

Importantly, an influence from the skin to the NIRS signal is not trivial. The NIRS devices used for clinical purposes provide light absorption into a depth of 3–4 cm. Extra-cranial tissue as indicated by dermal tissue flow, however, appears to contribute as much as 20% to rScO_2_, at least with the use of two commonly applied NIRS systems (Sørensen, pers. commun.). For estimation of muscle oxygenation light only needs to traverse skin and subcutaneous tissue that may be 2–3 mm thick (Kjeld et al., 2014) but subcutaneous tissue may, obviously be vast in obese patients. The penetration depth for light is proportional to the emitter-detector distance (Germon et al., 1999) of importance for light to reach brain tissue. Forehead skin is relatively thin in both adipose and lean patients, but the frontal sinuses in addition to the superior sagittal veins need to be considered (Sehic and Thomas, 2000). Also forehead skin blood flow is supplied with blood from both the internal and external carotid arteries (Hove et al., 2006) and with a headband preventing blood to enter the scalp, the rScO_2_ decreases (Davie and Grocott, 2012). This study clearly showed that three different NIRS devices weighed changes in skin flow differently of importance when NIRS is used to guide clinical interventions.

Vasopressor medication and its influence on NIRS deserve attention. Depending on the NIRS device used up to 1/3 of changes in rScO_2_ e.g., in response to administration of noradrenaline can be accounted for by change in skin blood flow (Sørensen et al., 2012). Thus, the INVOS cerebral oximeter appears more sensitive to changes in skin blood flow compared to the Foresight cerebral oximeter (Davie and Grocott, 2012). This could explain why ephedrine does not change rScO_2_ while strict α-adrenergic receptor stimulation such as treatment with norepinephrine (Brassard et al., 2009) or phenylephrine may decrease rScO_2_. In the case with hypotension causing cerebral deoxygenation, however, raised pressure with vasopressor medication may result in increased rScO_2_. When a low rScO_2_ is the combined effect of hypotension and lowered central blood volume, the use of α_1_-agonists such as phenylephrine may result in further cerebral desaturation due to a possible increase in cardiac afterload. Thus, a low cardiac output appears to influence CBF (van Lieshout et al., 2003) and phenylephrine might exert a different impact on cardiac output depending on preload to the heart (Cannesson et al., 2012). Furthermore, individual α- and β-adrenergic receptor sensitivity might be of importance and related to a genetic polymorphism (Snyder et al., 2006; Rokamp et al., 2013). When a vasopressor is administered the effect on rScO_2_ depends on individual factors and the NIRS technology used.

It remains that rScO_2_ responds to CO_2_ (Madsen and Secher, 1999) implying a contribution from the cerebrum since skin (and muscle) blood flow does not demonstrate “CO_2_ reactivity.” For clinical interventions directed to protect rScO_2_ it may, however, be less relevant whether the intervention is directed to address flow to the skin or the brain or both as long as the intervention improves postoperative outcome (Casati et al., 2005, 2007; Kazan et al., 2009; Slater et al., 2009; Papadopoulos et al., 2012; Stilo et al., 2012; Tang et al., 2012; Lin et al., 2013) including renal complications (Murkin et al., 2007) and wound infections (Ives et al., 2007). In addition, intraoperative severe cerebral desaturation may provoke postoperative vision loss (Pohl and Cullen, 2005; Roth, 2009). Thus, intraoperative rScO_2_ is an index for the systemic circulation reflecting changes in blood flow to other organs than the brain as the skin and kidney (Murkin and Arango, 2009).

Obviously, MAP should not be allowed to decrease to a level below the lower limit of cerebral autoregulation (60 mmHg). However, vasodilatation and reduction in intravascular volume challenge rScO_2_. While the spinal anesthesia induced vasodilatation causes only minor cerebral desaturation (Berlac and Rasmussen, 2005), the decrease in rScO_2_ is aggravated when hypotension is pronounced by the use of, e.g., hyperbaric bupivacaine (Kondo et al., 2013). On the other hand, the vasodilatation provoked by GA to minor surgery does not seem to affect rScO_2_ (Nissen et al., 2009a) may be because an effect on CBF is outweighed by a reduction in cerebral metabolism. In contrast, when GA is combined with procedures reducing cardiac output such as the anti-Trendelenburg body positions or the use of β-receptor antagonists, rScO_2_ decreases even at MAP at 80 mmHg (Lee et al., 2006).

**Figure 2:**
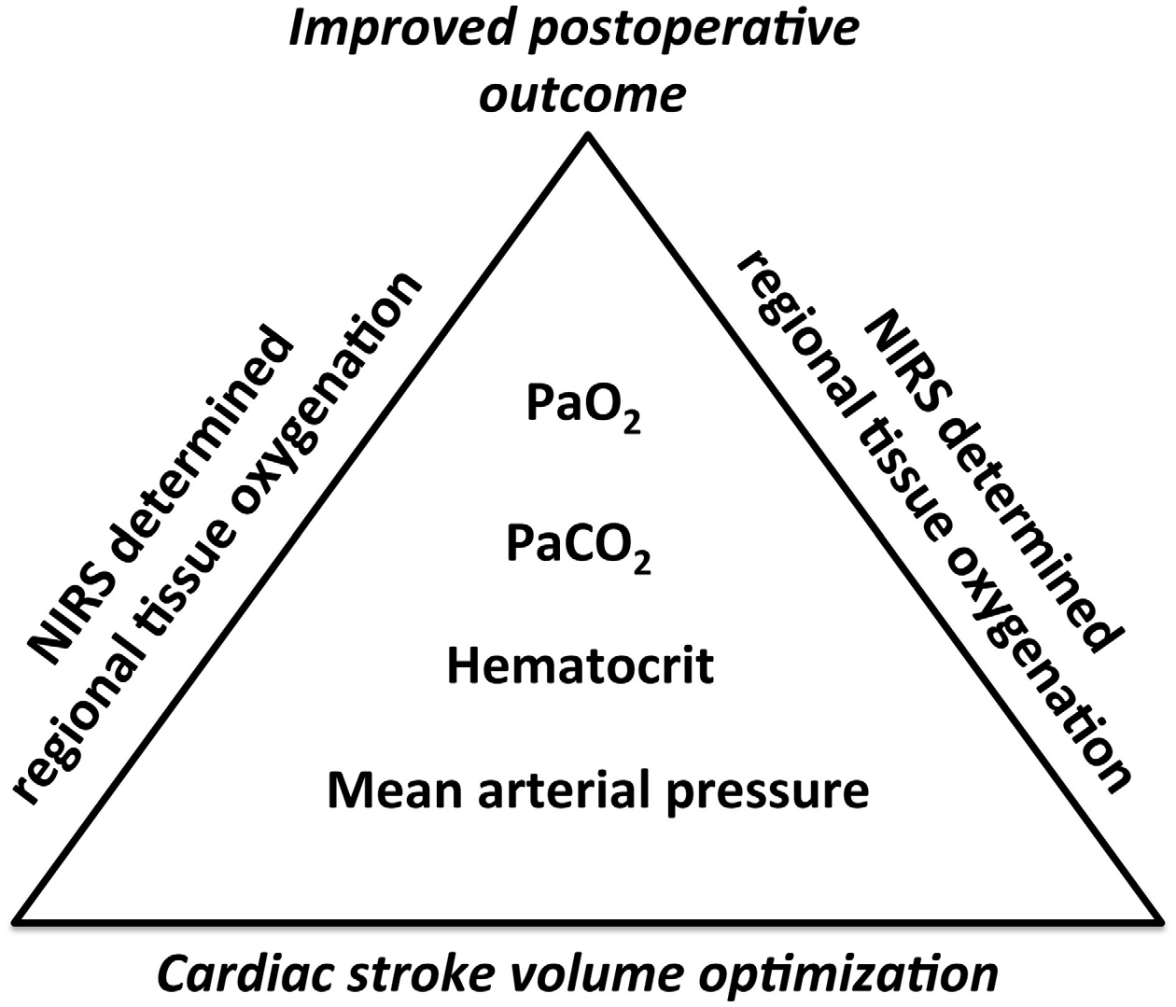
**A proposal for incorporation of near-infrared spectroscopy (NIRS) determined tissue in algorithms to maintain both central and peripheral blood flow in anesthesized patients.** Cardiac stroke volume is optimized by fluid administration and according to individual adjusted levels for mean arterial pressure (MAP), hematocrit, arterial carbon dioxide pressure (PaCO_2_), and arterial oxygen pressure (PaO_2_) it is secured that rScO2 does not change >11% considered the warning signal for postoperative complications (Kondo et al., 2013).

The majority of papers included in this review did not include a measurement of cardiac output but one study did find that rScO_2_ decreased 10% as cardiac output was reduced from 5 to 4 L/min (Lee et al., 2006). In addition, the use of phenylephrine reduces rScO_2_ secondary to a drop in cardiac output while ephedrine raises MAP without an effect on cardiac output (Meng et al., 2011). Thus, as mentioned vasopressors appear to affect rScO_2_ differently and before a vasopressor is used, it seems an advantage that the central blood volume is secured by optimization of, e.g., stroke volume or cardiac output by administration of fluid (Bundgaard-Nielsen et al., 2007b). Such so-called individualized goal directed fluid therapy reduces postoperative complications (Bundgaard-Nielsen et al., 2007a) as is the case for algorithms directed to maintain rScO_2_ (Casati et al., 2005; Murkin et al., 2007; Slater et al., 2009). Which of the two recommendations to manage circulation during anesthesia is most profitable remains to be evaluated, but the algorithms used to support the circulation could be combined as illustrated in Figure 2. Here it is recommended that management of a patients under GA includes not only NIRS monitoring of the brain but also a determination of cardiac output that can be derived easily, both non-invasively and invasively from the use of, e.g., model flow technology (van Lieshout et al., 2003).

In conclusion, this review on the use of NIRS to monitor changes in cerebral oxygenation of patients scheduled for non-cardiac surgery indicates that while rScO_2_ appears to be maintained in patients undergoing minor non-cardiac surgery such as mastectomy, rScO_2_ may decrease during surgery involving procedures such as the anti-Trendelenburg body position often used for shoulder surgery and laparoscopic surgery. Hip surgery, single lung ventilation in thoracic surgery, and clamped ICA also appear to be associated with a reduction of rScO_2_. An association of cerebral desaturation to postoperative outcome parameters such as acute kidney failure, postoperative wound infection, and myocardial infarction remains to be evaluated. After certain types of non-cardiac surgery severe cerebral desaturation might be associated with an increase in the occurrence of POCD.

### Conflict of interest statement

The author declares that the research was conducted in the absence of any commercial or financial relationships that could be construed as a potential conflict of interest.
